# Radiological Society of North America Expert Consensus Document on Reporting Chest CT Findings Related to COVID-19: Endorsed by the Society of Thoracic Radiology, the American College of Radiology, and RSNA

**DOI:** 10.1148/ryct.2020200152

**Published:** 2020-03-25

**Authors:** Scott Simpson, Fernando U. Kay, Suhny Abbara, Sanjeev Bhalla, Jonathan H. Chung, Michael Chung, Travis S. Henry, Jeffrey P. Kanne, Seth Kligerman, Jane P. Ko, Harold Litt

**Affiliations:** From the Department of Radiology, Perelman School of Medicine of the University of Pennsylvania, 3400 Spruce St, Philadelphia, PA 19104 (S.S., H.L.); Department of Radiology, University of Texas Southwestern Medical Center, Dallas, Tex (F.U.K., S.A.); Mallinckrodt Institute of Radiology, Washington University School of Medicine, St Louis, Mo (S.B.); Department of Radiology, University of Chicago Medicine, Chicago, Ill (J.H.C.); Department of Diagnostic, Molecular and Interventional Radiology, Icahn School of Medicine at Mount Sinai, New York, NY (M.C.); Department of Radiology and Biomedical Imaging, University of California San Francisco, San Francisco, Calif (T.S.H.); Department of Radiology, University of Wisconsin School of Medicine and Public Health, Madison, Wis (J.P.K.); Department of Radiology, University of California San Diego, San Diego, Calif (S.K.); and Department of Radiology, New York University Langone Health, New York, NY (J.P.K.).

## Abstract

Routine screening CT for the identification of coronavirus disease 19 (COVID-19) pneumonia is currently not recommended by most radiology societies. However, the number of CT examinations performed in persons under investigation for COVID-19 has increased. We also anticipate that some patients will have incidentally detected findings that could be attributable to COVID-19 pneumonia, requiring radiologists to decide whether or not to mention COVID-19 specifically as a differential diagnostic possibility. We aim to provide guidance to radiologists in reporting CT findings potentially attributable to COVID-19 pneumonia, including standardized language to reduce reporting variability when addressing the possibility of COVID-19. When typical or indeterminate features of COVID-19 pneumonia are present in endemic areas as an incidental finding, we recommend contacting the referring providers to discuss the likelihood of viral infection. These incidental findings do not necessarily need to be reported as COVID-19 pneumonia. In this setting, using the term viral pneumonia can be a reasonable and inclusive alternative. However, if one opts to use the term COVID-19 in the incidental setting, consider the provided standardized reporting language. In addition, practice patterns may vary, and this document is meant to serve as a guide. Consultation with clinical colleagues at each institution is suggested to establish a consensus reporting approach. The goal of this expert consensus is to help radiologists recognize findings of COVID-19 pneumonia and aid their communication with other health care providers, assisting management of patients during this pandemic.

Published under a CC BY 4.0 license.

## Introduction

Coronavirus disease 2019 (COVID-19) (1), caused by severe acute respiratory syndrome coronavirus 2 (SARS-CoV-2) (2), has become increasingly prevalent worldwide, reaching a pandemic stage in March 2020 (3). While most radiology professional organizations and societies have recommended against performing screening CT for the identification of COVID-19 (4,5), the number of CT examinations performed in persons under investigation (PUI) for COVID-19 may increase. We also anticipate that patients will have incidental lung findings on CT obtained for unrelated reasons that could be attributable to COVID-19.

Several recent publications have described CT imaging features of COVID-19, the evolution of these features over time, and the performance of radiologists in distinguishing COVID-19 from other viral infections (6–10). These studies have shown that COVID-19 often produces a CT pattern resembling organizing pneumonia, notably peripheral ground-glass opacities (GGOs) and nodular or masslike GGO that are often bilateral and multilobar (11). However, additional imaging findings have also been reported including linear, curvilinear, or perilobular opacities, consolidation, and diffuse GGO, which can mimic several disease processes including other infections, inhalational exposures, and drug toxicities (12–15).

COVID-19 pneumonia has a high mortality rate in some populations, including the elderly and those with diabetes, hypertension, and other comorbidities (16–18), and is spreading rapidly and sustainably in the community (19). As a result, including “COVID-19” in a radiology report could trigger a cascade of events including infection control measures and anxiety for both the managing provider and the patient. This potentially can complicate interpretations, as CT imaging features can overlap significantly with other causes of acute lung injury and organizing pneumonia (20). Standardized COVID-19 reporting language will improve communication with referring providers and has the potential to enhance efficiency and aid in management of patients during this pandemic.

This document aims to provide guidance to radiologists reporting CT findings potentially attributable to COVID-19 pneumonia in both PUI and when discovered incidentally. The potential role of CT in COVID-19; parameters for structured reporting; and the pros, cons, and limitations of adopting this strategy are discussed. In addition, practice patterns may vary by institution, and this document is meant to serve as a guide. If a radiologist, in accordance with one’s respective institutional procedures, chooses to mention COVID-19 specifically in CT reports, this standard framework can be adopted accordingly. Consultation with clinical colleagues at each institution is suggested to establish an agreed upon approach, which may evolve over time and be dependent upon the prevalence of the disease in the local population and other factors.

## Chest CT in COVID-19 Infection

### CT Imaging Features

Several papers have found that COVID-19 typically presents with GGO with or without consolidation in a peripheral, posterior, and diffuse or lower lung zone distribution (6–11). GGO has also been frequently reported to have round morphology or a “crazy paving” pattern (6,8). However, a significant portion of cases have opacities without a clear or specific distribution (8). A predominant perihilar pattern was not reported (8). Bronchial wall thickening, mucoid impactions, and nodules (“tree-in-bud” and centrilobular) seen commonly in infections, are not typically observed (8). Lymphadenopathy and pleural effusion have been rarely reported (6,21).

The frequency of imaging findings also depends on when infected patients are imaged. A slight majority of patients had a negative CT during the first 2 days after symptom onset with GGO usually developing between day 0 and 4 after symptom onset and peaking at 6–13 days (8,9,22–24). Therefore, a negative CT should not be used to exclude the possibility of COVID-19, particularly early in the disease. Later in the course of the disease, the frequency of consolidation increases as does the likelihood of seeing a reverse halo or atoll sign, typically absent near the time of symptom onset (8). Available evidence regarding these CT findings is limited, and new patterns of pulmonary involvement may eventually be reported (25).

### Diagnostic CT Performance and Screening

Chest CT findings can precede positivity on reverse-transcription polymerase chain reaction testing (RT-PCR). Early reports of RT-PCR sensitivity vary considerably, ranging from 42% to 71% (26,27), and an initially negative RT-PCR may take up to 4 days to convert in a patient with COVID-19 (26). The reported sensitivities and specificities of CT for COVID-19 vary widely (60%–98% and 25%–53%, respectively) (26–29), likely due to the retrospective nature of the currently published studies, including lack of strict diagnostic criteria for imaging and procedural differences for confirming infection. The positive and negative predictive value of chest CT for COVID-19 are estimated at 92% and 42%, respectively, in a population with high pretest probability for the disease (eg, 85% prevalence by RT-PCR) (27). The relatively low negative predictive value suggests that CT may not be valuable as a screening test for COVID-19 at least in earlier stages of the disease.

Literature comparing individual CT features of COVID-19 or radiologists’ performance in correctly choosing COVID-19 as a first-choice diagnosis on imaging is limited. In one study, six of seven radiologists demonstrated 93%–100% specificity in correctly distinguishing CT features of COVID-19 from other viral infections (10). A peripheral distribution of GGO was found to correctly distinguish COVID-19 from other viral causes 63%–80% of the time. However, the authors did not include high numbers of influenza-A or any noninfectious causes such as drug reaction, which could degrade radiologists’ performance.

### Viral Testing—Implications for CT

In reviewing CT publications on COVID-19, it is important to consider the accuracy of the laboratory viral testing used. This applies both to the collection method and the laboratory testing method (30), as many articles published on chest CT do not specify the sample collection or RT-PCR method used (31). With respect to collection method, bronchoalveolar lavage fluid (BALF) testing is the most sensitive, but not for general use given the invasive nature of fluid collection, and because it is an aerosol-generating procedure that could place health care workers at greater risk. Sputum and nasopharyngeal swab collection are considered equivalent in sensitivity, while throat swab testing is less sensitive. As viral pneumonias typically do not result in production of purulent sputum, nasopharyngeal swab is the preferred method for sample collection (31). As an example, in a recently published series of 1070 patients, the majority of samples collected were throat swabs, and throat swabs detected only approximately half of the positive cases that were detected by nasal swabs (32).

Rapid antigen tests are fast but have poor sensitivity. While RT-PCR is the most accurate, not all tests are equivalent. Eleven different RT-PCR tests were approved for use in China between January 26 and March 12, 2020, with varying levels of sensitivity. In a report of CT findings in 1014 patients (26), with 59% having a positive RT-PCR and 88% having a positive chest CT, the method of swab collection was not described. Two different RT-PCR tests were used, one of which does not appear on the list of approved tests, and the other approved for use in nasal, throat and sputum collection. The sensitivity of tests approved for use in the United States is high, with emergency use authorizations available on the website of the U.S. Food and Drug Administration (33).

### Structured Reporting

***Rationale and use.—***The goal of structured reporting in the setting of COVID-19 pneumonia is to help radiologists recognize the findings seen, decrease reporting variability, reduce uncertainty in reporting findings potentially attributable to this infection, and enhance the referring provider’s understanding of those radiologic findings, thereby allowing better integration into clinical decision making. While we do not currently recommend the use of CT screening for COVID-19 pneumonia, we suggest using a standardized language when specifically asked to address whether or not findings of COVID-19 pneumonia may be present on CT and propose language that could be placed in the impression of the report.

How to report incidentally discovered features potentially attributable to COVID-19 pneumonia is more complex. When typical features of COVID-19 pneumonia are present in an endemic area as an *incidental* finding, we recommend direct communication with the referring provider to discuss the likelihood of viral infection and to try to reach consensus. As always, radiologists should follow the ACR Practice Parameter for Communication of Diagnostic Imaging Findings (34). These incidental findings do not necessarily need to be reported as COVID-19 pneumonia, with “viral pneumonia” as a reasonable and inclusive alternative. However, if consensus is reached, and COVID-19 is mentioned as a potential diagnosis in the radiology report, we suggest using the provided standardized reporting language. Additionally, staff at the site performing the examination should be notified to initiate standard operating procedures (SOP) for potential exposure.

It should be noted that viral pneumonias have a wide variety of imaging manifestations, some of which are atypical or less common in COVID-19 such as tree-in-bud opacities and other small nodules, bronchial wall thickening, and bronchial mucus plugs (12). Thus, the term *viral pneumonia* encompasses a range of imaging findings some of which are not typical for COVID-19. It is also important to describe other lung abnormalities that may be associated with increased morbidity in the setting of COVID-19, such as emphysema and diffuse parenchymal lung disease.

***Categories.—***We propose four categories for reporting CT imaging findings potentially attributable to COVID-19, each with suggested standardized language (Table 1). The reporting language does not offer an exact likelihood for COVID-19 pneumonia, which depends on several factors including prevalence in a community, exposure, risk factors, and clinical presentation. Rather, the reporting language focuses on CT findings reported in the literature and the typicality of these features in COVID-19 pneumonia rather than other diseases. Included in the reporting language are unique coding identifiers in brackets that can then be used for future data mining.

**Table 1:**
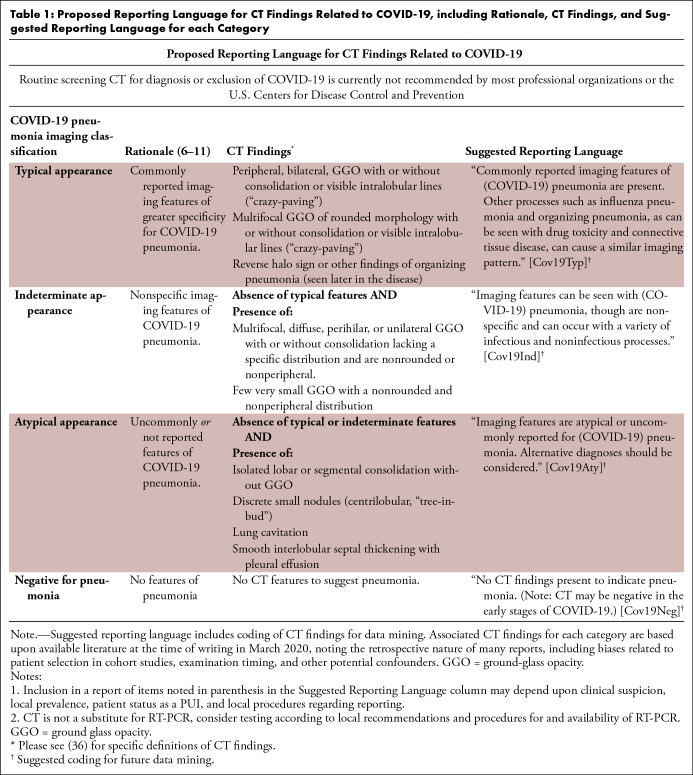
Proposed Reporting Language for CT Findings Related to COVID-19, including Rationale, CT Findings, and Suggested Reporting Language for each Category

**Figure 1:**
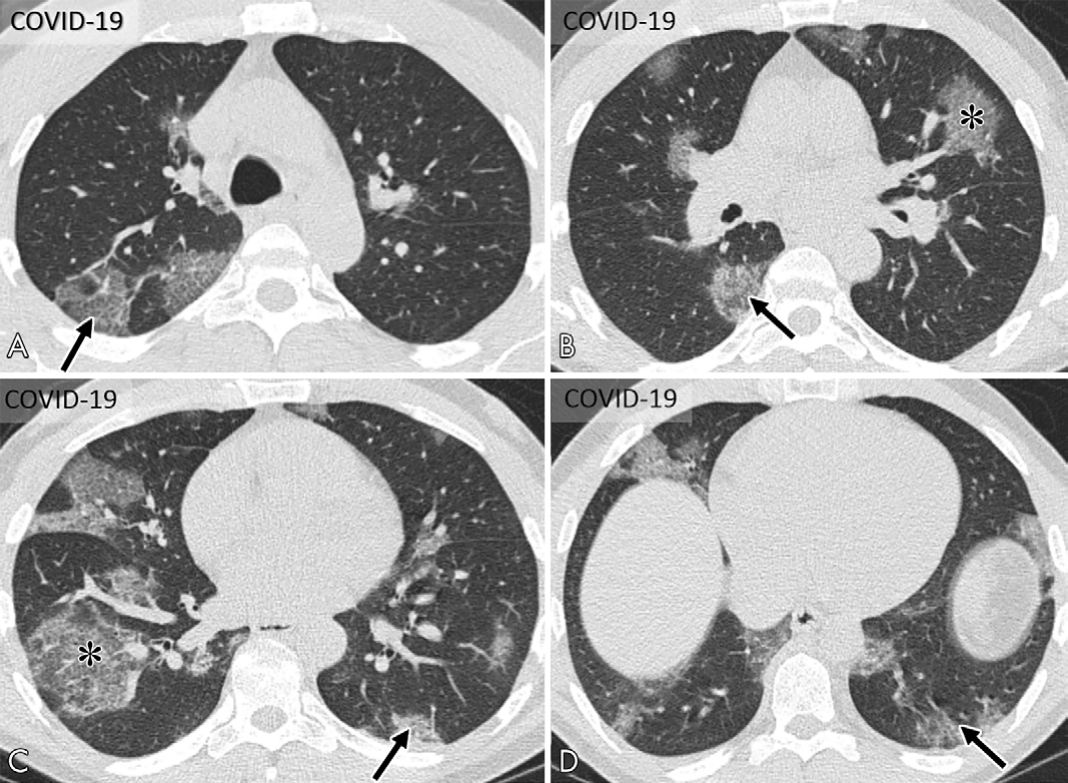
Typical CT imaging features for COVID-19. Unenhanced thin-section axial images of the lungs in a 52-year-old man with a positive RT-PCR (*A–D*) show bilateral, multifocal rounded (asterisks) and peripheral GGO (arrows) with superimposed interlobular septal thickening and visible intralobular lines (“crazy-paving”). Routine screening CT for diagnosis or exclusion of COVID-19 is currently not recommended by most professional organizations or the U.S. Centers for Disease Control and Prevention.

**Figure 2:**
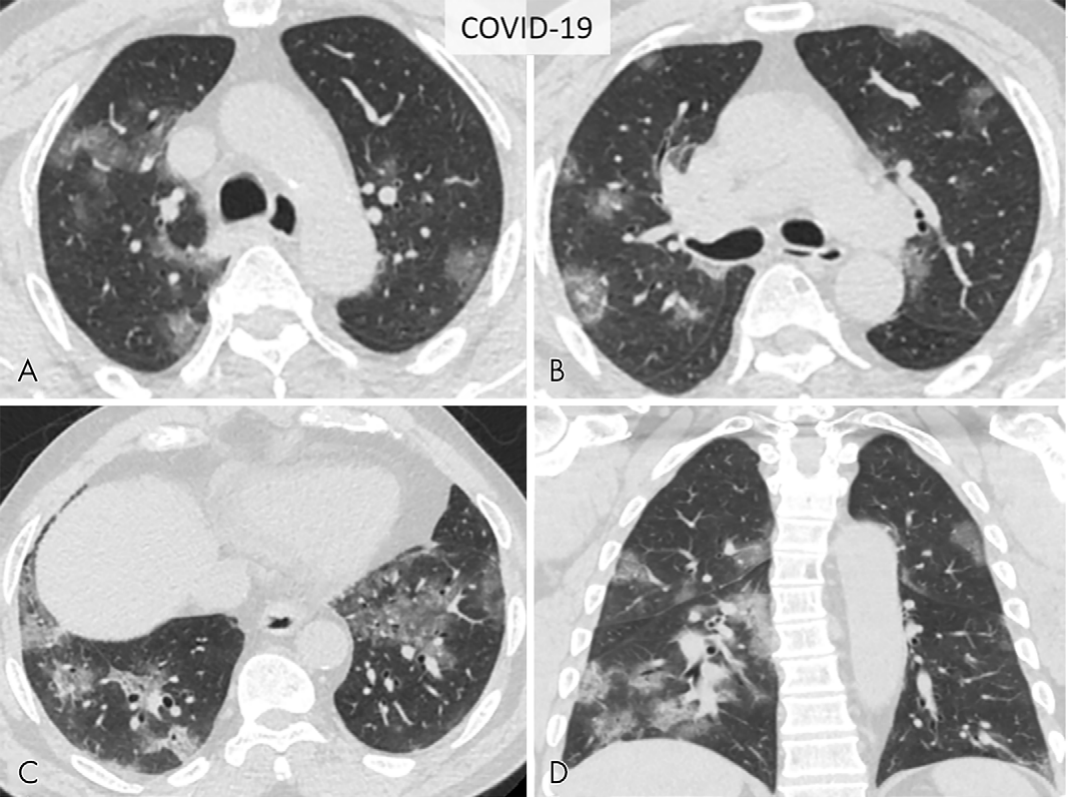
Typical CT imaging features for COVID-19. Unenhanced thin-section axial (*A–C*) and coronal multiplanar reformatted images (*D*) of the lungs in a 77-year-old man with a positive RT-PCR show bilateral, multifocal rounded and peripheral GGO. Routine screening CT for diagnosis or exclusion of COVID-19 is currently not recommended by most professional organizations or the U.S. Centers for Disease Control and Prevention.

**Figure 3:**
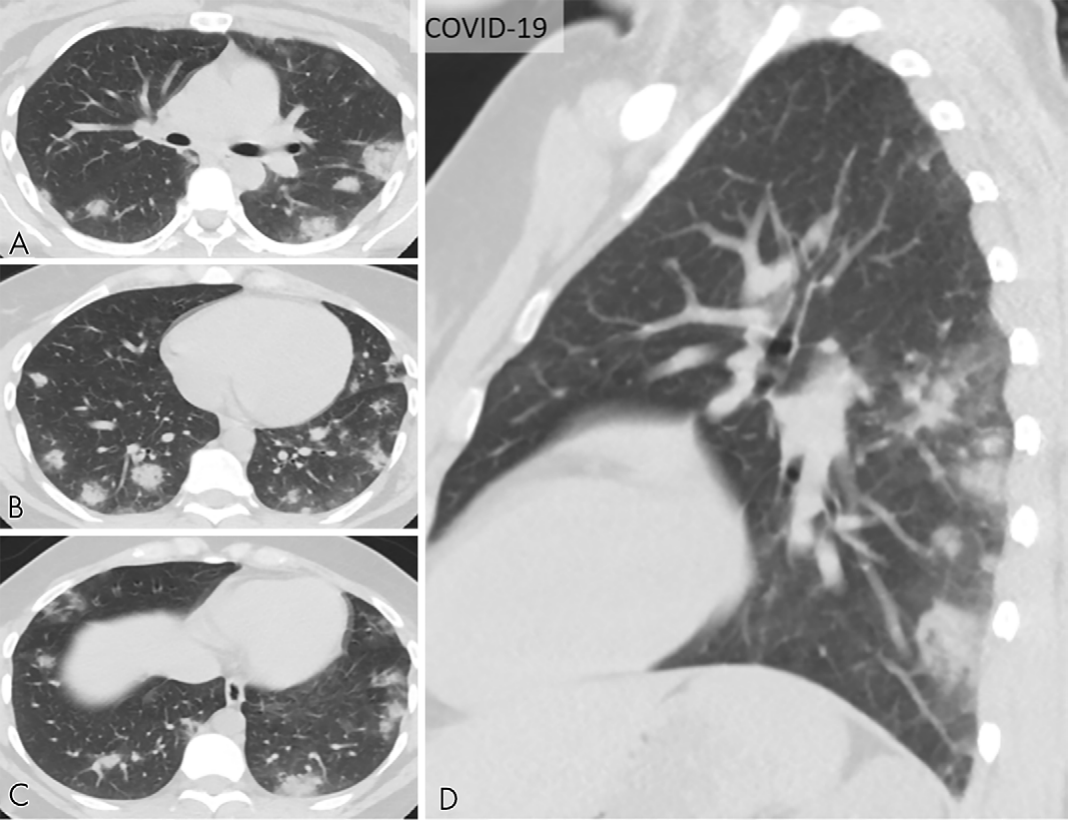
Typical CT imaging features for COVID-19. Unenhanced axial (*A–C*) and sagittal multiplanar reformatted (*D*) images of the lungs in a 29-year-old man with a positive RT-PCR show multiple bilateral, rounded consolidations with surrounding GGO. Routine screening CT for diagnosis or exclusion of COVID-19 is currently not recommended by most professional organizations or the U.S. Centers for Disease Control and Prevention.

**Figure 4:**
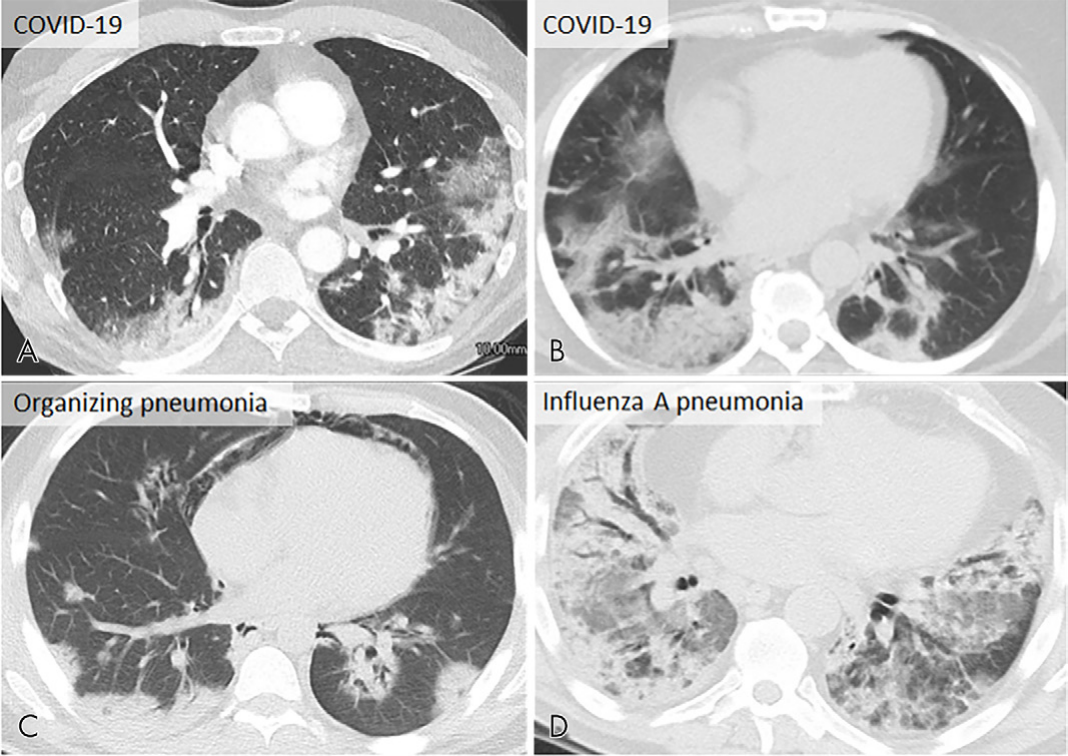
Typical CT imaging features for COVID-19 and other diseases with similar findings. Posterior, peripheral, and rounded GGO and consolidation in axial images of four patients; COVID-19 (*A, B*), organizing pneumonia secondary to dermatomyositis (*C*), and influenza A pneumonia (*D*). Organizing pneumonia and influenza pneumonia can be indistinguishable from COVID-19 by CT. Routine screening CT for diagnosis or exclusion of COVID-19 is currently not recommended by most professional organizations or the U.S. Centers for Disease Control and Prevention.

Typical features are those that are reported in the literature to be frequently and more specifically seen in COVID-19 pneumonia in the current pandemic (10,11). (Figs 1–4). The principal differential diagnosis includes some viral pneumonias, especially influenza, and acute lung injury patterns, particularly organizing pneumonia, either secondary, such as from drug toxicity and connective tissue disease, or idiopathic.

**Figure 5:**
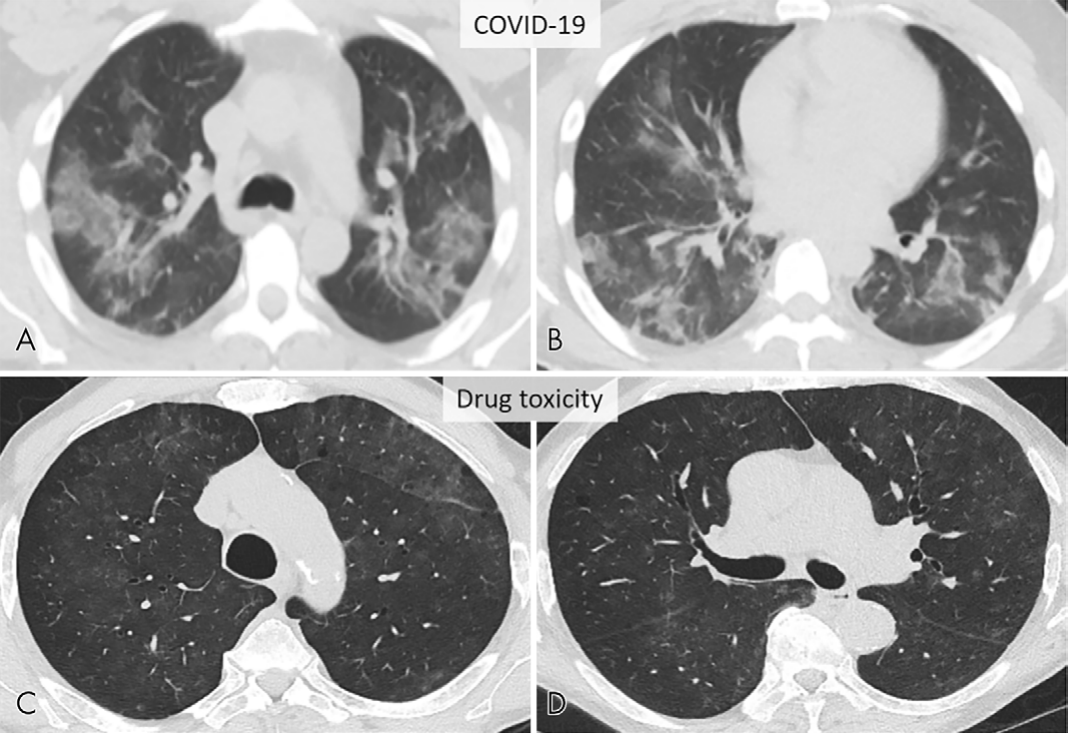
Indeterminate CT imaging features for COVID-19. Unenhanced axial images in two patients showing patchy GGO with nonrounded morphology and no specific distribution, in a case of COVID-19 pneumonia (*A, B*) and acute lung injury from presumed drug toxicity (*C, D*). Routine screening CT for diagnosis or exclusion of COVID-19 is currently not recommended by most professional organizations or the U.S. Centers for Disease Control and Prevention.

**Figure 6:**
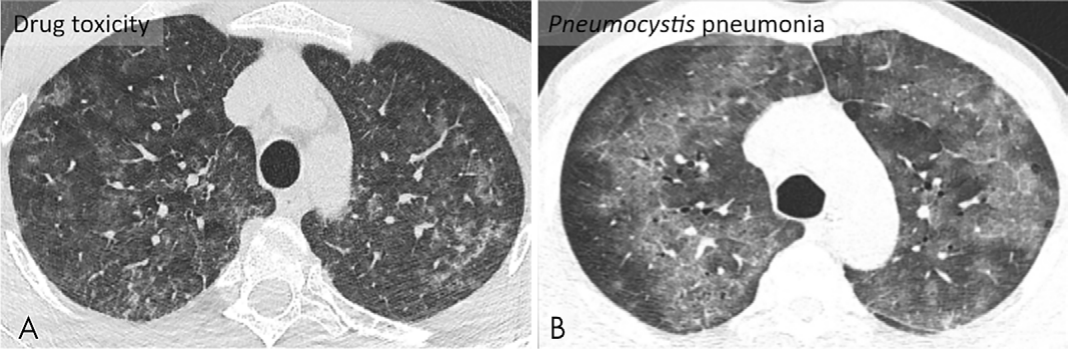
Indeterminate CT imaging features for COVID-19. Widespread GGO with nonrounded morphology and no specific distribution in unenhanced axial images from two different patients secondary to acute lung injury from presumed drug toxicity (*A*) and *Pneumocystis* pneumonia (*B*). Routine screening CT for diagnosis or exclusion of COVID-19 is currently not recommended by most professional organizations or the U.S. Centers for Disease Control and Prevention.

Indeterminate features are those that have been reported in COVID-19 pneumonia but are not specific enough to arrive at a relatively confident radiologic diagnosis. An example would be diffuse GGO without a clear distribution (Figs 5, 6). This finding is common in COVID-19 pneumonia but occurs in a wide variety of diseases such as acute hypersensitivity pneumonitis, *Pneumocystis* infection, and diffuse alveolar hemorrhage, which are difficult to distinguish by imaging alone.

**Figure 7:**
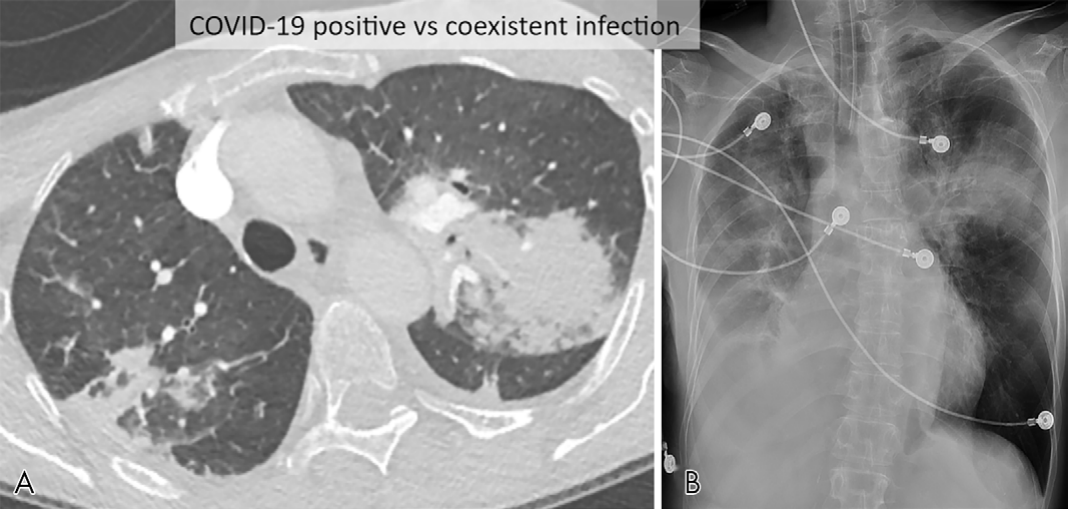
Atypical CT imaging features for COVID-19. Contrast-enhanced axial CT image (*A*) and frontal chest radiograph (*B*) showing segmental consolidation without significant GGO. Although this patient tested positive for COVID-19, the imaging features are not typical and could represent pneumonia related to COVID-19 or a secondary infectious process. Routine screening CT for diagnosis or exclusion of COVID-19 is currently not recommended by most professional organizations or the U.S. Centers for Disease Control and Prevention.

**Figure 8:**
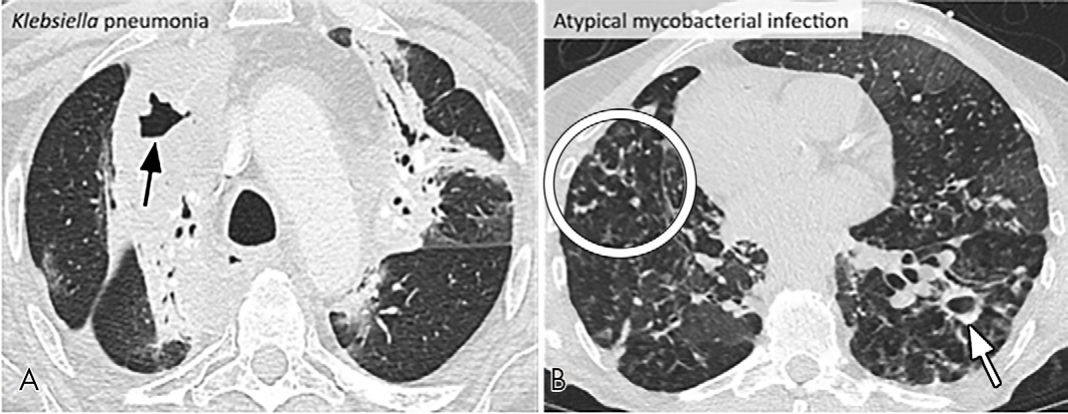
Atypical CT imaging features for COVID-19. Axial images of the lungs of two patients showing cavitation (arrow) in *Klebsiella* pneumonia (*A*) and tree-and-bud opacities (circle) and a cavity (arrow) in nontuberculous mycobacterial infection (*B*). Routine screening CT for diagnosis or exclusion of COVID-19 is currently not recommended by most professional organizations or the U.S. Centers for Disease Control and Prevention.

**Figure 9:**
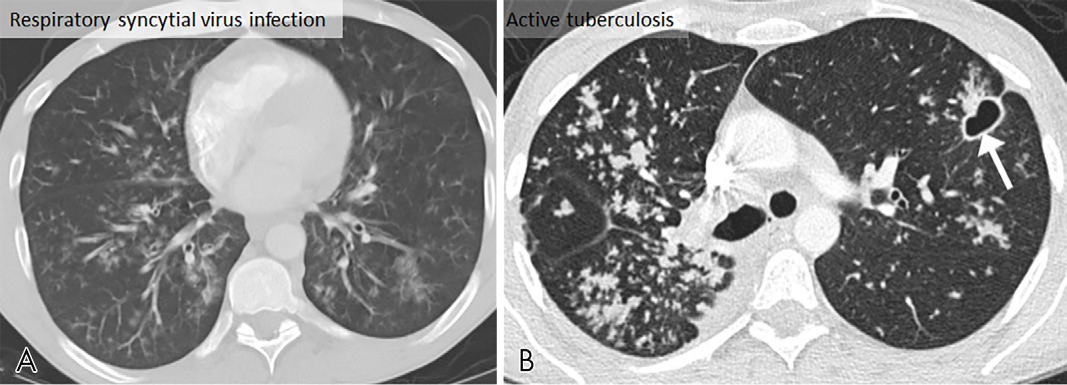
Atypical CT imaging features for COVID-19. Axial CT images from two different patients showing tree-in-bud opacities and centrilobular nodules, caused by respiratory syncytial virus (*A*) and active tuberculosis (*B*). A small cavity (arrow) is also present in (*B*) Routine screening CT for diagnosis or exclusion of COVID-19 is currently not recommended by most professional organizations or the U.S. Centers for Disease Control and Prevention.

Atypical features are those that are reported to be uncommon or not occurring in COVID-19 pneumonia and are more typical of other diseases such as lobar or segmental consolidation in the setting of a bacterial pneumonia, cavitation from necrotizing pneumonia, and tree-in-bud opacities with centrilobular nodules, as can occur with a variety of community acquired infections and aspiration (Figs 7–9).

Negative for pneumonia implies that there are no parenchymal abnormalities that could be attributable to infection. Specifically, GGO and consolidation are absent. Importantly, there may be no findings on CT early in COVID-19. Conversely, CT has been reported to be more sensitive than RT-PCR earlier in the course of the disease (29), although this result may change with local RT-PCR test characteristics.

### Pros, Cons, and Limitations of Standardized Reporting

There are compelling arguments both for and against the use of standardized reporting language in describing CT findings potentially attributable to COVID-19 (Table 2). This project largely stemmed from the expectation that despite most current professional guidelines recommending against the routine use of screening CT for COVID-19, CT may be requested for potential assistance in diagnosis and management, particularly if RT-PCR is not readily available.

**Table 2:**
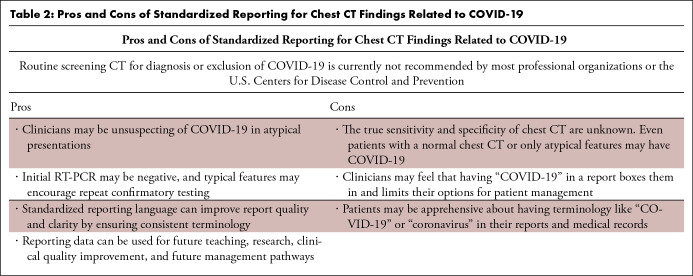
Pros and Cons of Standardized Reporting for Chest CT Findings Related to COVID-19

***Pros.—***Without expert consensus, radiologists may be left with uncertainty as to how to convey the presence, absence, or likelihood of COVID-19 when confronted with this as a specific indication or as an incidental finding. Standardized reporting can provide guidance and confidence to radiologists as well as increased clarity to providers through reduced reporting variability. Providing unique identifiers for each category facilitates mining data for future educational, research, and quality improvement. In addition, standardized radiology reports combined with clinical assessment may enable future care pathways to determine which patients may preferentially undergo RT-PCR should testing capacity be exceeded. Initial RT-PCR testing may also be negative, and typical imaging findings may encourage repeat testing.

***Cons.—***The true sensitivity and specificity of CT for COVID-19 remains relatively unknown. One study showed that radiologists identified COVID-19 versus other viral pneumonias correctly 60%–83% of the time based on typical CT imaging features (10). However, the results of this study must be evaluated cautiously as all of the COVID-19 cases came from one country (China) and most of the control cases from a single institution in another country (United States). Additionally, this moderate level of distinction may be reduced in clinical practice as the control cases included a low proportion of influenza-A, which is the major viral pneumonia that must be differentiated from COVID-19 during the winter and spring months across the northern hemisphere. Reporting “atypical features” may result in false-negative cases, and the risk of missing COVID-19 can have broad implications. Ordering providers may also feel that having “COVID-19” or “coronavirus” documented in a radiology report constrains their clinical decision making and treatment options. This concern is less relevant in PUIs, as clinical suspicion already exists. However, difficulties may arise in patients with findings suggestive of COVID-19 that are incidentally detected. Direct communication with the referring provider about the likelihood of COVID-19 is recommended to avoid surprising providers and patients. We again emphasize that as an incidental finding, particularly with indeterminate or atypical features, “viral pneumonia” may be preferable to “COVID-19” or “coronavirus.”

***Limitations.—***We anticipate cases with mixed imaging findings, that is, those that have both typical and atypical imaging features for COVID-19. Recent analysis suggests that over 20% of patients with COVID-19 may have coexistent infections complicating the categorization of imaging observations (35). The radiologist will have to determine whether or not these findings are part of the same process or are unrelated. For example, a hospitalized patient undergoing chest CT for fever could have lower lobe tree-in-bud opacities as well as peripheral GGO, which could reflect aspiration superimposed on viral pneumonia. It is also possible that atypical features such as lobar consolidation may reflect a secondary bacterial pneumonia even in patients who test positive for COVID-19. Available evidence is still limited concerning the appearance of COVID-19 in the presence of secondary disease processes such as coexistent infections and aspiration. In scenarios such as these, discussion with the treating team would be prudent.

Imaging appearances in the standardized reporting language are based upon available literature at the time of writing in March 2020, noting the retrospective nature of many reports, including biases related to patient selection in cohort studies, examination timing, and other potential confounders. As radiologists’ experience with COVID-19 increases, our categorization of these findings as typical, indeterminate, or atypical may evolve.

## Conclusions

We propose four categories for the suggested standardized CT reporting language of COVID-19 based on current literature and expert consensus. We acknowledge that for patients with unexpected findings that could be attributed to COVID-19, the matter is complex and that “viral pneumonia” is a reasonable alternative. As always, radiologists should follow the ACR Practice Parameters for Communication of Diagnostic Imaging Findings. If COVID-19 is a potential incidental diagnosis, staff at the site performing the examination should be notified to initiate SOP for potential exposure. We also acknowledge that practice patterns vary, and this document is intended to provide guidance. If a radiologist chooses to mention COVID-19 in CT reports, this is a standard framework that can be adopted. Consensus between local imaging and clinical providers is essential to establish an agreed-upon approach.

At this time, CT screening for the detection of COVID-19 is not recommended by most radiologic societies. However, we anticipate that the use of CT in clinical management as well as incidental findings potentially attributable to COVID-19 will evolve. We believe it important to provide radiologists and referring providers guidance and confidence in reporting these findings and a more consistent framework to improve clarity. Clear and frequent communication among health care providers, including radiologists, is imperative to improving patient care during this pandemic.
